# Impact of adjuvant chemotherapy on outcomes in appendiceal cancer

**DOI:** 10.1002/cam4.3009

**Published:** 2020-03-19

**Authors:** Bhaskar C. Kolla, Ashley Petersen, Madhuri Chengappa, Tulasi Gummadi, Chitra Ganesan, Wolfgang B. Gaertner, Anne Blaes

**Affiliations:** ^1^ Division of Hematology, Oncology and Transplantation University of Minnesota Minneapolis MN USA; ^2^ Division of Biostatistics University of Minnesota Minneapolis MN USA; ^3^ Department of Medicine University of Minnesota Minneapolis MN USA; ^4^ Department of Surgery University of Minnesota Minneapolis MN USA; ^5^ Division of Hematology, Oncology and Transplantation University of Minnesota Minneapolis MN USA; ^6^ Present address: GME Internal Medicine Department Nazareth Hospital 2601 Holme Avenue Philadelphia PA 19152 USA; ^7^ Present address: North Memorial Health Cancer Center 3435 W Broadway Ave W Robbinsdale MN 55422 USA; ^8^ Present address: Frauenshuh Cancer Center 3931 Louisiana Ave. S St Louis Park MN 55426 USA

**Keywords:** adjuvant chemotherapy, appendiceal cancer, impact of chemotherapy, overall survival, progression‐free survival

## Abstract

**Background:**

The impact of using adjuvant chemotherapy following cytoreductive surgery (CRS) and hyperthermic intraperitoneal chemotherapy (HIPEC) in patients with appendiceal adenocarcinoma is not known. The aim of this study was to assess the impact of adjuvant chemotherapy following complete cytoreduction in patients with appendiceal adenocarcinoma.

**Methods:**

Retrospective medical record review of all patients with appendiceal adenocarcinoma treated at our institution between 2006 and 2015. Kaplan‐Meier plots were used to summarize overall survival (OS) and relapse‐free survival over time, and log‐rank tests and Cox proportional hazards models were used to test for differences in survival between groups.

**Results:**

A total of 103 patients with appendiceal adenocarcinoma received care at our institution during the study period. Complete cytoreduction (cytoreductive score 0‐1) was achieved in 68 patients (66%). Of these 68 patients, 26 received adjuvant chemotherapy. The most common regimens were capecitabine (n = 11), capecitabine plus oxaliplatin (n = 7), and 5‐FU plus oxaliplatin (n = 6). Tumor histopathology and grade, and the ability to achieve complete cytoreduction were significant predictors of overall survival. The median OS for non–low‐grade and well‐differentiated tumor patients who received adjuvant chemotherapy following complete cytoreduction was 9.03 years, compared to 2.88 years for patients who did not receive adjuvant chemotherapy (*P* = .02). Among low‐grade and well‐differentiated tumor patients who underwent complete cytoreduction, there was no statistically significant difference in OS between those who received adjuvant chemotherapy and those who did not.

**Conclusion:**

Adjuvant chemotherapy seems to have benefit in appendiceal cancer patients with non–low‐grade or well‐differentiated tumor type but not in low‐grade or well‐differentiated tumors.

## Graphical abstract

The impact of adjuvant chemotherapy following complete cytoreduction in various histopathological subtypes of appendiceal cancer remains unknown. This retrospective study shows that adjuvant chemotherapy has benefit only in moderate‐ to high‐grade and signet‐ring type adenocarcinomas but not in low‐grade and well‐differentiated appendiceal adenocarcinomas.

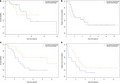

## 1 INTRODUCTION

Appendiceal cancer is a heterogeneous entity ranging from low‐grade mucinous tumors, which typically cause mechanical problems from mucin accumulation such as bowel obstruction; to aggressive high‐grade tumors with poor differentiation and signet‐ring cell morphologies. Cytoreductive surgery (CRS) has a significant role in the management of appendiceal neoplasms.^1^ Systemic chemotherapy has also been shown to have benefit.^2^, ^3^ However, its impact when given in the perioperative setting is not known. Given the scarcity of these tumors, conducting randomized studies to understand the impact of chemotherapy is difficult.

However, there are multiple retrospective studies that examined the role of chemotherapy. Systemic chemotherapy has been shown to have little to no effect in low‐grade and well‐differentiated appendiceal adenocarcinomas. Earlier studies from a small group of 17 patients with pseudomyxoma peritonei (PMP) from Smith JW et al in 1992 reported no benefit from adjuvant chemotherapy on overall survival (OS) following cytoreductive surgery and suggested systemic chemotherapy be reserved for patients with recurrent disease.^4^ Gough DB in 1994 reported adverse outcomes with use of any systemic chemotherapy in patients with PMP.^5^ Recently, Lu et al also reported similar findings.^6^ They reported systemic chemotherapy had no effect on OS in a large National Cancer Database (NCDB) study of 639 patients with stage IV low‐grade mucinous appendiceal adenocarcinoma.^6^ Conversely, systemic chemotherapy has been shown to have beneficial impact in moderate‐ to high‐grade mucinous tumors, signet‐ring cell tumors, and all nonmucinous tumors. Shapiro et al^3^ and Lieu et al^7^ reported systemic chemotherapy has activity in patients with unresectable disease or high‐grade and signet‐ring tumors, respectively. In the latter study by Lieu et al, adjuvant chemotherapy was of borderline significance on relapse‐free survival (RFS) and OS in high‐grade and signet‐ring tumors. Similarly, systemic chemotherapy was also beneficial in 109 patients with stage IV nonmucinous appendiceal carcinoma, all of whom had either moderate or poorly differentiated histologies.^8^ Only one prospective study was conducted evaluating impact of preoperative systemic chemotherapy, which showed improved OS in high‐grade peritoneal carcinomatosis with signet‐ring cell features with use of neoadjuvant chemotherapy.^9^ In a very large population dataset, Asare et al in 2016 reported improvement in OS with systemic chemotherapy in stage IV patients with all nonmucinous tumors and moderate and poorly differentiated mucinous tumors.^2^ There was no significant benefit for well‐differentiated mucinous tumors.^2^ The majority of these studies reported the impact of systemic chemotherapy in stage IV patients, but the data on impact of adjuvant chemotherapy following complete cytoreduction in stage IV patients are limited.

Guidelines per National Comprehensive Cancer Network (NCCN) state that data are quite limited for appendiceal adenocarcinoma and advises practitioners to consider chemotherapy per NCCN guidelines for colon cancer.^10^ A review of current clinical practice at NCCN member institutions showed practitioners commonly use colorectal cancer data.^11^ However, there are recent studies that showed molecular differences between appendiceal and colorectal cancer,^12^, ^13^ indicating a different biology and potentially different response to similar treatments. The extrapolation of data from colorectal cancer studies to guide chemotherapeutic choices in appendiceal cancer may be misleading.

Our objective was to determine whether there was a difference in OS or RFS between patients who received adjuvant chemotherapy following complete cytoreductive surgery and those who did not.

## 2 METHODS

All patients with appendiceal carcinoma who were treated at our institution between January 2006 and December 2015 were included. A retrospective chart review was performed to extract all necessary demographic and clinical information for our analysis. Histopathology, imaging, and operative reports were reviewed to identify the accurate histopathological diagnosis, grade, and stage of disease as well as completeness of cytoreduction and cytoreductive score (CC). Complete cytoreduction was defined as surgical resection with no macroscopic residual disease (CC‐0) or residual tumor deposits less than 2.5 mm (CC‐1). Incomplete cytoreduction includes any residual tumor with tumor deposits more than 2.5 mm. Adjuvant chemotherapy was defined as systemic chemotherapy administered following complete cytoreduction, with intention to delay or prevent relapse. All patients who are diagnosed at an outside institution underwent pathology review by an expert GI pathologist at our institution. The Peritoneal Surface Oncology Group International (PSOGI) consensus terminology was used for histopathological classification^14^ and AJCC 2017 (eighth edition) TNM staging was used for tumor staging. Due to natural heterogeneity, with multiple histopathological subtypes and grades of appendiceal neoplasms, and resultant small sample size in each group, comparison of outcomes between each individual group would be challenging. Prior studies showed benefit for systemic chemotherapy only in moderate‐to‐poorly differentiated mucinous tumors, signet‐ring cell tumors, and all nonmucinous tumors, but not in low‐grade and well‐differentiated mucinous tumors. To effectively analyze and compare outcomes, we grouped all tumors into two groups based on their reported responsiveness or lack of responsiveness to systemic chemotherapy: (a) Low‐grade and well‐differentiated tumors and (b) Non–low‐grade and well‐differentiated tumors. The low‐grade and well‐differentiated tumor group included low‐grade mucinous neoplasms (LAMNs) and well‐differentiated mucinous adenocarcinoma. The non–low‐grade and well‐differentiated tumor group included all other tumors. Institutional review board approval was obtained. Only adults 18 years or above were included. Patients with neuroendocrine and goblet cell histopathologies were excluded. Overall survival is defined as time elapsed since date of diagnosis to date of death. Relapse‐free survival is defined as time elapsed since complete cytoreduction surgery to radiological or biopsy‐proven relapsed disease or death.

All CRS/HIPEC procedures were performed by two surgeons at a single center that performs over 40 HIPEC procedures per year. All operations are preceded by or started with a diagnostic laparoscopy in order to determine resectability. If the Peritoneal Cancer Index (PCI) was <20, there was no diffuse small bowel involvement, and no high‐risk findings such as pelvic tumor involvement requiring an exenterative procedure, large‐volume ascites, diffuse retroperitoneal involvement, tumor encasement of the pancreas, duodenum, or portal vein, and bilobar liver metastases; CRS was performed with the intention of obtaining a CC 0‐1, with subsequent HIPEC using a closed technique. Mitomycin C (20 mg per BSA m^2^) was uniformly distributed throughout the peritoneal cavity for 90 minutes at mean temperature of 40°C.

### 2.1 Statistical analysis

Descriptive statistics were used to summarize demographic and clinical information. Means and standard deviations were used for continuous variables and counts and percentages were used for categorical variables. Kaplan‐Meier plots were used to summarize OS and RFS over time, and log‐rank tests and Cox proportional hazards models were used to test for differences in survival between groups. All statistical testing was performed at the 0.05 level. All statistical analyses were performed using R version 3.4.1 with the ‘survival’ package.^15^

## 3 RESULTS

There were a total of 103 patients with appendiceal cancer treated at our institution between 2006 and 2015, with adequate records for review of treatment and outcomes (Table 1). Mean age was 54 years and 58% of patients were women. A majority of patients (83%) were stage 4 at diagnosis. Ninety‐three patients underwent surgery with complete cytoreductive intent. Peritoneal cancer index was 0 in 13 patients, between 1 and 9 in 26 patients, 10‐19 in 23 patients, >20 in 32 patients, and unknown in nine patients. Complete cytoreduction (CC‐0&1) was amenable in 68 patients (66%). Of these, 26 patients (39%) received adjuvant chemotherapy. The most common regimens were capecitabine (n = 11), capecitabine plus oxaliplatin (n = 7), and 5‐FU plus oxaliplatin (n = 6).

**TABLE 1:** Clinicopathologic characteristics

	Overall (n = 103)	Incomplete cytoreduction or no surgery (n = 35)	Complete cytoreduction (n = 68)
Age at diagnosis (y), mean (SD)	54 (12)	56 (12)	53 (12)
Male	43 (42%)	18 (51%)	25 (37%)
Stage at diagnosis			
1	2 (1.9%)	0 (0%)	2 (2.9%)
2	9 (8.7%)	0 (0%)	9 (13%)
3	6 (5.8%)	0 (0%)	6 (8.8%)
4	86 (83%)	35 (100%)	51 (75%)
Histopathology and grade			
Low‐grade mucinous neoplasm	8 (7.8%)	2 (5.7%)	6 (8.8%)
Mucinous adenocarcinoma			
Well‐differentiated	38 (37%)	11 (31%)	27 (40%)
Moderately differentiated	16 (16%)	7 (20%)	9 (13%)
Poorly differentiated	3 (2.9%)	0 (0%)	3 (4.4%)
Grade unknown or treatment effect	3 (2.9%)	1 (2.9%)	2 (2.9%)
Adenocarcinoma with signet‐ring cell features (<50% of tumor)	13 (13%)	7 (20%)	6 (8.8%)
Signet‐ring cell adenocarcinoma	15 (15%)	5 (14%)	10 (15%)
Nonmucinous adenocarcinoma			
Well‐differentiated	0 (0%)	0 (0%)	0 (0%)
Moderately differentiated	5 (4.9%)	1 (2.9%)	4 (5.9%)
Poorly differentiated	2 (1.9%)	1 (2.9%)	1 (1.5%)

Abbreviation: SD, standard deviation.

**FIGURE 1:**
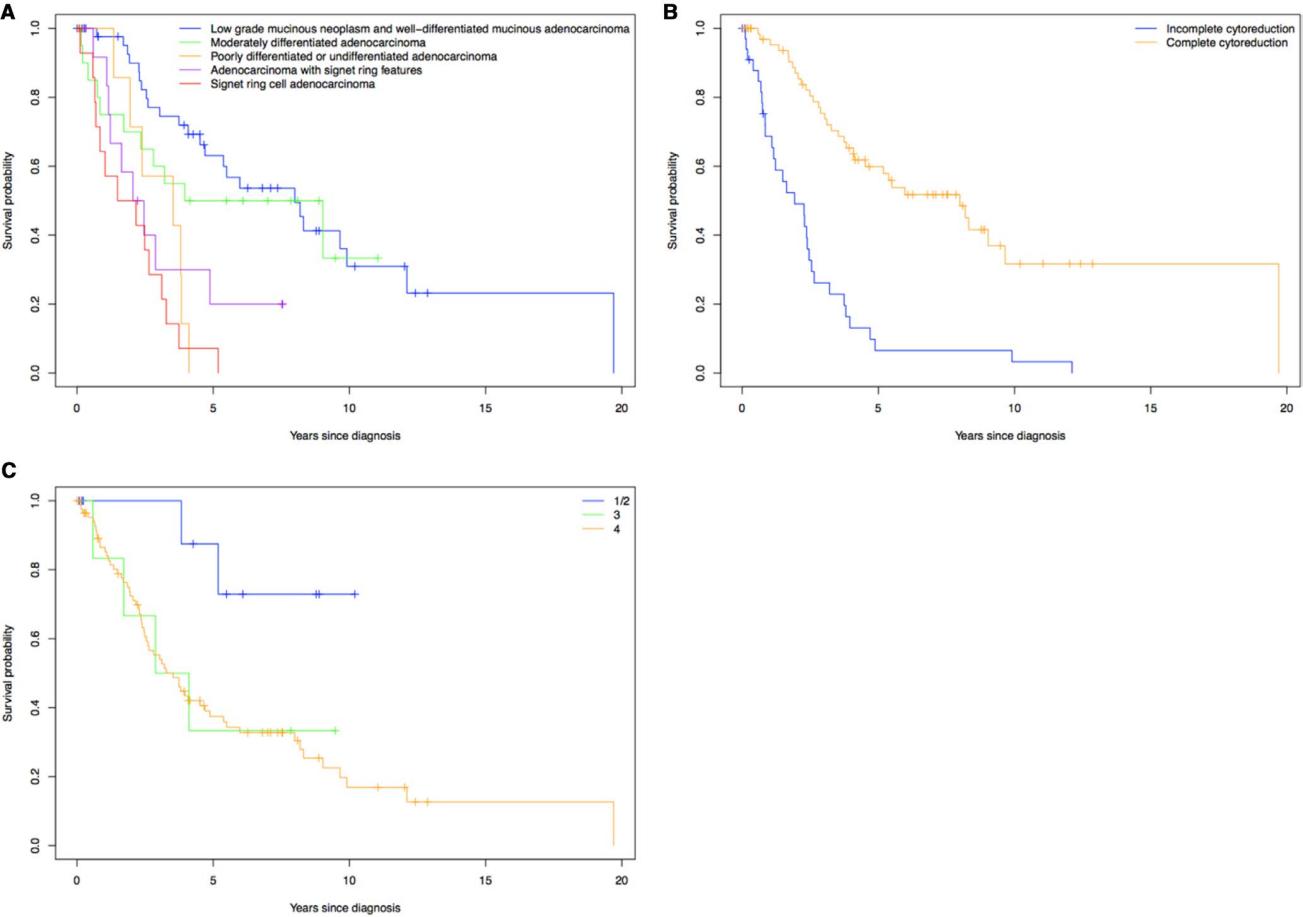
Overall survival based on (A) histopathology and grade, (B) complete vs incomplete cytoreduction, and (C) stage at diagnosis

The impact of histopathology, complete cytoreduction, and stage at diagnosis on OS are outlined in Table 2. Patients with low‐grade mucinous neoplasm and well‐differentiated adenocarcinoma had the longest median OS (7.99 [4.70, ‐] years), whereas those with signet‐ring cell adenocarcinoma had the shortest median OS (1.83 [0.84, 3.74] years; Figure 1A). Complete cytoreduction (n = 68) resulted in a median OS of 7.99 years, compared to 1.93 years for patients with incomplete cytoreduction (n = 35, Figure 1B). There was a trend toward stage at diagnosis being significantly associated with OS (*P* = .09), but the limited number of early stage patients (n = 11 who were stage 1 or 2 at diagnosis) limited this comparison (Figure 1C).

**TABLE 2:** Impact of histopathology, complete cytoreduction, and stage at diagnosis

	n	Median OS in years (95% CI)^a^, ^b^
Effect of histopathology		*P* < .001
Low‐grade mucinous neoplasm and well‐differentiated mucinous adenocarcinoma	46	7.99 (4.70, ‐)
Moderately differentiated adenocarcinoma (both mucinous and nonmucinous)	21	6.49 (2.35, ‐)
Poorly differentiated or undifferentiated adenocarcinoma (both mucinous and nonmucinous)	8	3.53 (1.95, ‐)
Adenocarcinoma with signet‐ring features (<50%)	13	2.25 (1.22, ‐)
Signet‐ring cell adenocarcinoma.	15	1.83 (0.84, 3.74)
Effect of complete cytoreduction		*P* < .001
Incomplete cytoreduction (CC‐2&3)	35	1.93 (1.16, 2.64)
Complete cytoreduction (CC‐0&1)	68	7.99 (4.52, ‐)
Stage at diagnosis		*P* = .09
1 or 2	11	‐ (5.18, ‐)
3	6	3.49 (1.71, ‐)
4	86	3.53 (2.55, 5.38)

Abbreviations: CC, cytoreductive score; CI, confidence interval; OS, overall survival.

^a^ The *P*‐values are from a Cox proportional hazards model with these predictors.

^b^ Omitted values were not estimable due to censoring.

**FIGURE 2:**
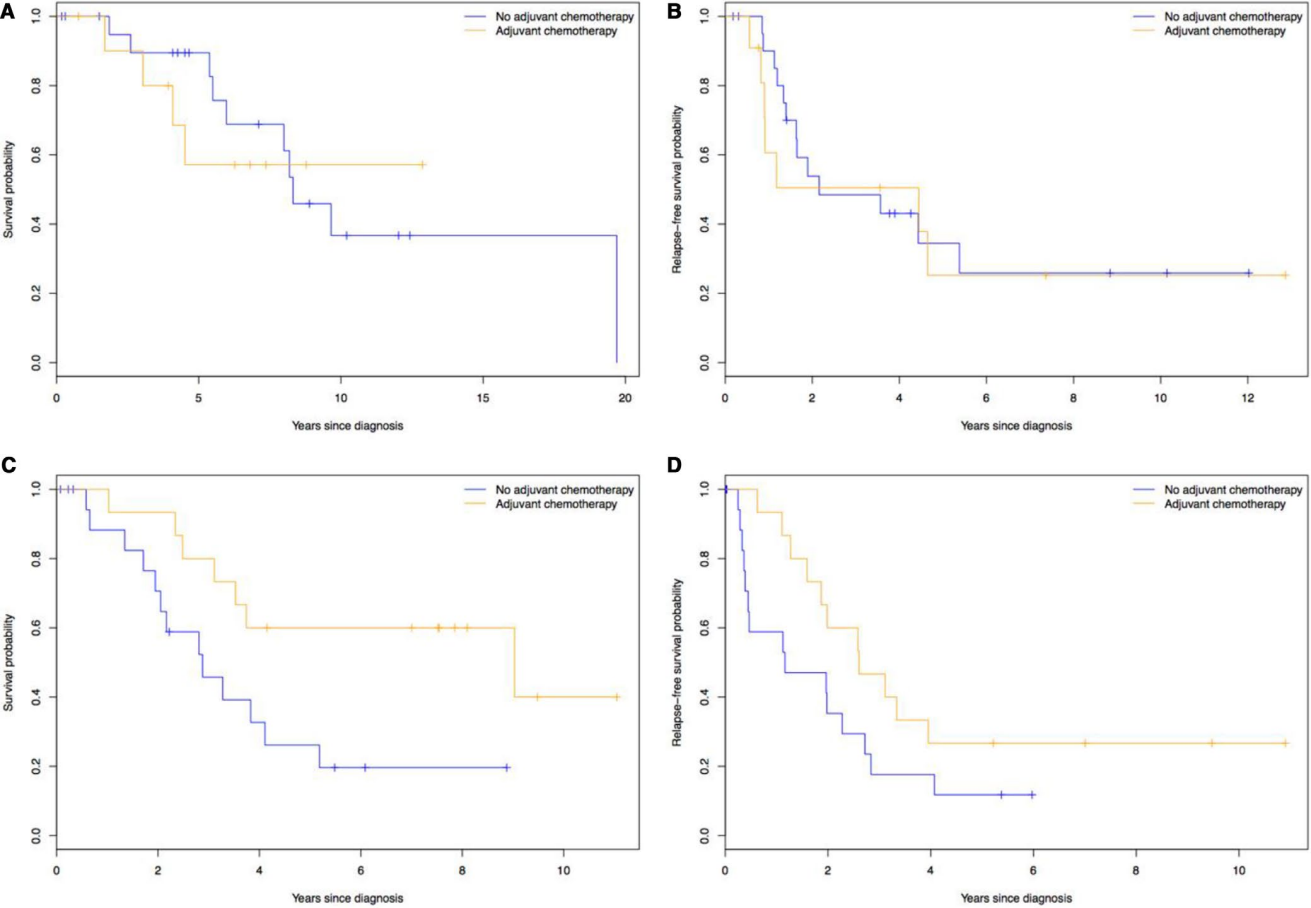
Differential impact of adjuvant chemotherapy in low‐grade or well‐differentiated tumors for (A) overall survival and (B) relapse‐free survival and non–low‐grade or well‐differentiated tumors for (C) overall survival and (D) relapse‐free survival

The impact of adjuvant chemotherapy following complete cytoreduction is outlined in Table 3. Adjuvant chemotherapy following complete cytoreduction resulted in longer OS in non–low‐grade or well‐differentiated tumor patients compared with patients who did not receive chemotherapy (median 9.03 vs 2.88 years, *P* = .02, Figure 2C). Additionally, there was a trend for longer RFS in the adjuvant chemotherapy group compared with patients who did not receive chemotherapy (median 2.60 vs 1.16 years, *P* = .09, Figure 2D). Neither OS nor RFS were significantly different between adjuvant chemotherapy and no adjuvant chemotherapy groups in patients with low‐grade and well‐differentiated tumors (Figure 2A,B).

**TABLE 3:** Impact of adjuvant chemotherapy following complete cytoreduction

	n	Median OS in years (95% CI)^a^, ^b^	Median RFS in years (95% CI)^a^, ^b^
Effect of adjuvant chemotherapy after complete cytoreduction in low‐grade or well‐differentiated adenocarcinoma		*P* = .80	*P* = .73
No adjuvant chemotherapy	22	8.32 (5.98, ‐)	2.16 (1.63, ‐)
Adjuvant chemotherapy	11	‐ (4.08, ‐)	4.45 (0.90, ‐)
Effect of adjuvant chemotherapy after complete cytoreduction in non–low‐grade or well‐differentiated adenocarcinoma		*P* = .02	*P* = .09
No adjuvant chemotherapy	20	2.88 (2.05, ‐)	1.16 (0.45, 2.84)
Adjuvant chemotherapy	15	9.03 (3.53, ‐)	2.60 (1.87, ‐)

Abbreviations: CI, confidence interval; OS, overall survival; RFS, relapse‐free survival.

^a^ The *P*‐values given are from a log‐rank test.

^b^ Omitted values were not estimable due to censoring.

## 4 DISCUSSION

Given that appendiceal cancer is a very heterogenous entity, we analyzed the impact of adjuvant chemotherapy based on low‐grade/well‐differentiated tumors *vs* non–low‐grade/well‐differentiated tumors. Prior studies suggested a differential benefit for systemic chemotherapy in patients with moderate‐ and high‐grade mucinous tumors but not for low‐grade mucinous tumors.^2^ We found similar differential benefit with adjuvant chemotherapy in this study. Among patients who underwent complete cytoreduction, adjuvant chemotherapy was associated with longer median OS only in the moderate‐to‐poorly differentiated and signet‐ring adenocarcinoma patients but not in patients with low‐grade mucinous neoplasm or well‐differentiated adenocarcinoma.

Eighty three percent of patients in our study population were stage 4 at diagnosis, compared to 45% of patients in a large National Cancer Data Base (NCDB) study reported by Asare et al^2^ We also had a higher percentage of patients (15%) with signet‐ring cell adenocarcinoma compared to 9% reported in the NCDB study. This is likely due to a high number of patients in our study group who were referred to our institution for a second opinion regarding cytoreductive surgery after primary tumor resection at an outside hospital. This likely resulted in a larger proportion of tumors with poor biology and advanced stage. The majority of patients with mucinous type tumors were of low‐grade or well‐differentiated histology, whereas all of our patients with nonmucinous adenocarcinoma were either moderately or poorly differentiated. This was similar in distribution to the previously described NCDB study.

Overall survival of patients in each histological category is comparable with other studies in the literature.^16^, ^17^, ^18^ There is great heterogeneity in tumor histopathology and grade, with survival largely dependent on these two variables. The benefit of complete cytoreduction, which has been established previously, was noted again in our study.^1^ Patients who achieved complete cytoreduction had a median survival of 7.99 years compared to 1.93 years for those unamenable to complete cytoreduction. This probably reflects an inherent selection bias, with aggressive tumors likely presenting with more extensive and locally invasive disease making them unamenable for complete cytoreduction.

Our study is limited by its retrospective nature and single institution experience; hence, the findings may not be generalizable to a larger population. The study population as well as appendiceal cancers, in general, are a heterogeneous group with a small number of patients in each subset. Hence, the subset analysis based on individual histopathological groups is infeasible in any single institutional study given the rarity of these tumors. Despite these limitations, the study provides evidence that adjuvant chemotherapy following complete CRS may be most beneficial in those with non–low‐grade or well‐differentiated tumors.

## 5 CONCLUSION

Adjuvant chemotherapy following complete cytoreductive surgery seems to have significant benefit in overall survival in patients with moderate‐ to high‐grade and signet‐ring cell appendiceal tumors. Complete cytoreduction significantly influences oncologic outcomes in patients with appendiceal neoplasms. A large multi‐institutional study is necessary to further analyze outcomes by each individual category.

## CONFLICT OF INTEREST

None of the authors report any relevant conflict of interest.

## AUTHOR CONTRIBUTIONS

All authors contributed significantly to the conceptualization, data gathering, and analysis. BCK, AP, WBG, and AB prepared manuscript and all authors approved the final version of the manuscript.

## Data Availability

The data that support the findings of this study are available from the corresponding author upon reasonable request.
